# Research Priorities for Malignant Pleural Organization with Loculation and Failed Drainage

**DOI:** 10.3390/cells14141118

**Published:** 2025-07-21

**Authors:** Torry A. Tucker, Erminia Massarelli, Luis Destarac, Steven Idell

**Affiliations:** Department of Cellular and Molecular Biology, Center for Biomedical Research, School of Medicine, The University of Texas Health Science Center at Tyler, Tyler, TX 75708, USAerminia.massarelli@uthct.edu (E.M.); luis.destarac@uthealth.com (L.D.)

**Keywords:** malignant pleural effusions, loculation, IPFT/IET: Intrapleural fibrinolytic or enzymatic therapy

## Abstract

Malignant pleural effusion (MPE) can lead to pleural organization with loculation and impaired drainage. This condition is becoming increasingly more common due to advancements in cancer therapy and extended patient survival. Factors such as repeated thoracentesis through an indwelling pleural catheter (IPC), intrapleural bleeding, and tumor progression contribute to MPE organization. Loculated MPE causes breathlessness and reduced quality of life, and current therapies, including intrapleural fibrinolytic or enzymatic therapy (IPFT/IET), have limitations in efficacy and safety. Identifying new therapeutic targets is crucial for improving treatment outcomes. Research is needed to understand the role of profibrogenic factors in pleural neoplasia, their regulation, and their impact on different stages of pleural organization. The development of a rabbit model of organizing MPE could provide insights into underlying mechanisms and novel interventions. Comparative studies of pleural tissues and effusions from MPE patients and other forms of pleural organization may reveal valuable information. Cellular and molecular profiling, assessment of biomarkers, and personalized IPFT dosing are potential areas of investigation. Suppression of PAI-1 activity and the role of hyaluronic acid in malignant mesothelioma are also important research directions. Understanding the profibrogenic capacity of pleural mesothelial cells undergoing mesenchymal transition (MesoMT) and identifying key contributors and effectors involved in this process are essential for developing effective treatments for loculated MPE.

## 1. Introduction

**Figure 1:**
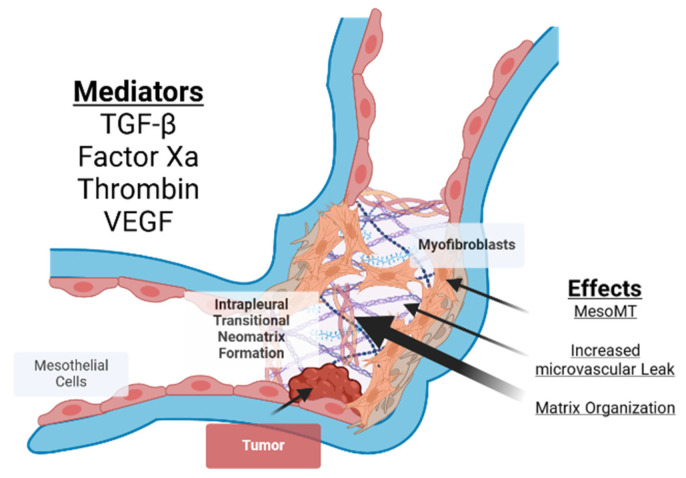
Organization of MPEs. Tumor progression can induce vascular leakage, because of cytokines such as VEGF, resulting in the accumulation of MPE. Visceral (**top**) and parietal (**bottom**) pleural surfaces are indicated in blue and lined by mesothelial cells. These effusions subsequently undergo coagulation, leading to the formation of a fibrinous intrapleural transitional neomatrix. If these effusions persist, they can undergo organization with loculation leading to fibrotic entrapment of the lung in MPE. A range of mediators have been implicated in these processes and major contributors are illustrated. Among these, TGF-β, thrombin and coagulation factor Xa can cause resident pleural mesothelial cells to transition into profibrotic myofibroblasts through a process known as mesothelial mesenchymal transition (MesoMT).

Pleural organization can lead to loculation, which may complicate malignant pleural effusion (MPE). This situation is frequently seen in current medical practice and likely will increase with improved cancer therapy and extended survival of patients with advanced disease [1,2]. Pleurodesis, sclerosant-induced obliteration of the pleural space, and intrapleural catheter placement are commonly used to control excessive intrapleural fluid in MPE. Pleural organization within MPE fluid collections can alternatively impair pleural fluid drainage and decrease lung function in patients with MPE [3,4,5,6,7,8,9,10]. Pleurodesis involves intrapleural administration of a sclerosant—talc, tetracyclines, chemotherapeutic or other agents—to induce inflammation and fibrosis of the pleural surfaces [10,11]. An indwelling pleural catheter (IPC) can alternatively be used to control MPE-induced dyspnea in patients with MPE. Loculation of MPE can be initiated by repeated thoracentesis or repetitive aspiration of MPE via IPC [12] bleeding [7,13,14], complicating empyema [15] while trapped lung can derive from progression of intrapleural tumors that form rind at the pleural surfaces [16,17] (Figure 1). Loculated MPE can likewise cause breathlessness and reduced quality of life. Management of loculated MPE represents an independent, important clinical challenge. Current therapy of loculated MPE is problematic, particularly since many patients cannot tolerate surgical relief because of their advanced malignancy and comorbidities. Intrapleural fibrinolytic (or enzymatic) therapy (IPFT/IET) is advocated to expedite pleural drainage and relieve dyspnea in these patients, but uncertainty persists about its efficacy, dosing, choice of agents, and how best to best mitigate bleeding risks [18]. The clinical presentation and imaging of loculated MPE resembles that of other forms of pleural organization, including empyema, retained hemothorax, particularly in the sense that all may involve loculation, and impairment of pleural drainage [19]. Identifying new therapeutic targets is necessary and represents a significant gap in the field, especially those that may be most specific to MPE.

## 2. The Pathogenesis of Tissue Organization and Fibrosis

Organization connotes a cardinal, sequential pathophysiologic process whereby tissue fibrosis occurs, which first involves the development of microvascular leakage, followed by extravascular coagulation with deposition of a transitional fibrinous matrix and subsequent progressive fibrosis. There has been long-standing appreciation of the importance of these events and their contributions to the pathogenesis of inflammation, neoplasia, and wound healing [20]. The process occurs in several different organs including the lungs and may occur in the response of tissues to malignancies with associated desmoplasia [21,22]. Within the pleural compartment, progressive organization appears to lead to pleural loculation with impaired drainage and fibrosis including the development of fibrothorax or trapped lung with desmoplasia involving the pleural surfaces in some cases of MPE [19] (Figure 1).

To date, aberrant fibrin turnover with formation of transitional matrix has led to actionable treatments that are primarily directed to enhancing fibrinolysis to clear pleural loculation, enhance drainage and improve outcomes. The inception of this approach was first advocated about seventy years ago [23,24]. Since then, intrapleural fibrinolytic therapy (IPFT has widely been used to expedite pleural drainage in a range of clinical settings [18,19]. The approach has involved the use of plasminogen activators to enhance conversion of intrapleural fibrinolysis and thereby clear loculations. Recently, the use of plasminogen supplementation has alternatively been proposed to drive fibrinolysis and achieve the same results, although clinical testing of this concept requires further investigation [25].

Tissue factor is mainly responsible for the induction of the extrinsic coagulation pathway within the pleural compartment, leading to activation of downstream coagulation factors such as factor Xa or thrombin [19]. These activated coagulation factors exceed potential inhibition by locally elaborated tissue factor pathway inhibitor (TFPI) and favor formation of the transitional fibrinous collections that form pleural loculations. We found that intrapleural with or without intravenous administration of site inactivated factor VII or TFPI were unable to clear loculations in a rabbit model of intrapleural loculation induced by tetracycline (unpublished data). Interestingly, either intrapleural heparin or urokinase blocked adhesion formation in the same model [26]. We are unaware of any clinical trials which have tested the ability of intrapleural anticoagulants that have been used to treat loculated MPE or improve drainage nor in any other clinical form of pleural organization. The availability of potent new factor Xa or thrombin inhibitors to attenuate loculation in preclinical models or clinical trials has not, likewise, been evaluated to our knowledge. In addition, we know of no testing of TGF-β or TNF-α inhibitors to attenuate experimental or clinical forms of pleural loculation to date.

A similar sequence of events has long been attributed to the pathogenesis of pleurodesis [27,28]. The administration of sclerosants including tetracycline or other agents induces secretion of proinflammatory and profibrogenic mediators, including TGF-β and VEGF, which promote pleural fluid accumulation and angiogenesis. Mesothelial cells are believed to be central to the elaboration of these mediators in the pathogenesis of pleurodesis and pleural loculation in MPE [29]. While these processes involve common proinflammatory and profibrotic derangements, they can occur in different locales. Obliteration of the pleural space can control pleural fluid accumulation and relieve dyspnea in MPE, but pleural loculation can sequester pleural fluid, prevent drainage and promote dyspnea even with placement of an IPC in MPE.

Other processes may contribute to the pathogenesis of pleural loculation, although the precise pathways remain to be defined. Novel ways of profiling pleural fluids have been applied, which have yielded new potential biomarkers. For example, metabolomic profiling of pleural fluids identified arginine as a candidate biomarker able to distinguish MPE from parapneumonic or tuberculous pleural effusions [30]. In the future, a multiomic range of analyses may augment cellular DNA profiling to expand our understanding of mesothelial cell mesenchymal plasticity in the pathogenesis of loculation, as reviewed previously [31]. In another screening approach, DNA methylation analyses of pleural fluids have been performed to assess epigenetic changes associated with MPE [32]. These analytic strategies have yet to be applied to define how newly recognized mediator or epigenetic changes affect loculation in MPE, but they represent potentially rewarding opportunities for future translational study.

## 3. Organization in MPE and Other Forms of Pleural Loculation

**Figure 2:**
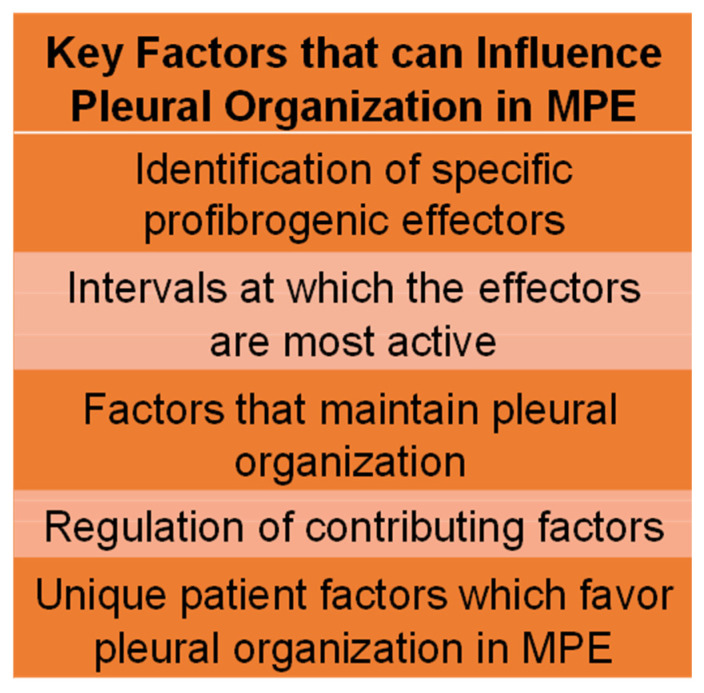
Key Factors That May Influence Organization of Malignant Pleural Effusion (MPE). There is currently a paucity of knowledge regarding how these effectors and factors operate in control of the onset and persistence of pleural organization in MPE.

Organization of pleural tissues with the development of intrapleural loculation may occur in MPE, as in retained hemothorax or pleural infections [19], suggesting that common factors underlie this endpoint. On the other hand, the definition of new specific effectors that contribute to organization of MPEs and how and when they operate in the process remain to be clarified (Figure 2). The time course of clinical pleural loculation and pleural thickening after the development of MPE may differ in each of these scenarios and from patient to patient, possibly based on genetic differences or other contributing factors that likewise remain to be defined (Figure 2). In a similar vein, some patients with pleural infections may not be loculated but have loose pleural septations, while others may loculate shortly after presentation or be loculated with impaired pleural drainage and dyspnea at presentation. Variation in the presence of loculation versus “free-flowing” pleural effusions is also suggested by the distribution of such patients in the MIST-2 trial, where most but not all patients with pleural infection were loculated [33]. The dose of tPA used in this trial was empirically selected and to our knowledge, there are no forms of IPFT which are approved by the FDA or any other regulatory body. There have been no dose response phase 1 trials to assess any form of IPFT with the exception of the dose-ranging testing of single-chain urokinase [34]. This agent has not yet been approved by the FDA and all currently available forms of IPFT have been likewise used off-label at empirically selected doses and dosing schedules. In empyema, retained hemothorax and MPE, coagulation substrates are present with concurrent derangements that impair local fibrinolysis, as previously reviewed [19,35] (Figure 1). The appearance of transitional fibrin typically occurs within the pleural compartment accompanying pleural inflammation that culminates in formation of pleural adhesions, simulating loculation in preclinical models, as previously reviewed. Similar derangements of fibrin may occur in MPE [19]. In empyema and retained hemothorax associated with trauma, the presence of pleural inflammation and coagulation may promote formation of transitional fibrinous matrices and loculation. Exudative MPEs exhibit similar derangements associated with inflammation following repeated thoracentesis in patients with MPE [36], which may initiate loculation, impaired pleural drainage and breathlessness. Whether there are specific derangements in coagulation, fibrinolytic or other profibrogenic pathways in different forms of MPE is now unknown and in our view, worthy of further study.

## 4. Incidence of and Management of Loculated MPE

The incidence of pleural loculation and failed drainage in MPE is now unclear and there is a dearth of past or present literature addressing the topic [37]. The presence of adhesions in MPE has been suggested to be indicative of poor prognosis [38]. Despite the paucity of incidence data, loculation of MPE is now commonly managed in clinical practice, so that the scope of the problem appears to be broad. It is likely that the incidence of MPE will continue to increase commensurate with improvements in oncologic interventions and increased survival of patients with advanced stage cancer. Effective pleural drainage management strategies are a key priority, and these include several modalities in addition to control of pleural fluid burden and dyspnea with pleurodesis [39]. Among these, IPFT/IET is commonly used in patients with loculated MPE to mitigate the need for surgical intervention, particularly in inoperable patients with stage 4 cancer and pleural involvement with loculation and failed pleural drainage [6,37,40]. Urokinase-based IPFT does not appear to increase the success of pleurodesis in loculated MPE with impaired drainage, but length of hospital stay was shortened, pleural opacification reduced and short-term (1-year) survival versus placebo-treated patients increased [41]. On the other hand, quality of life and dyspnea scores were not improved. In patients with loculation and an IPC, instillation of IPFT with no DNase improved pleural drainage and dyspnea, but recurrence of symptomatic loculation and dyspnea were observed [42]. The single dose IPFT treatment format in this study raises the question about the predicate for such dosing. Whether multiple dose IPFT therapy is safe and more effective is now unclear and requires further investigation. At present, the use of IPFT has recently been advocated to improve pleural drainage in patients with loculated MPE particularly with an IPC and limited alternative options [40]. Further studies are needed to clarify how IPFT should be delivered to ensure the best possible clinical outcomes for patients with MPE, loculation, failed drainage and dyspnea.

## 5. Low Dose IPFT and IPC Delivery

Because it is well-recognized that IPFT can be complicated by bleeding, attempts to reduce its risk by lowering the dose of the fibrinolytic agent have been reported. A few case reports suggest that low dose tPA delivered through an IPC may relieve dyspnea in patients with symptomatic loculated MPE [9,43], but the broad applicability of this approach is unclear. Whether the use of adjuncts improves outcomes is also unclear, but a range of IPFT doses of tPA (2.5–10 mg) combined with 5 mg DNase was used to safely and effectively treat IPC-related pleural loculation and reduce the need for surgery [44]. There are no preclinical studies of which we are aware that address the dosing range of IPFT that is safe and most effective, and this customary guide to clinical trial safety design is now lacking. The available studies suggest that there may be an optimal lower dose, perhaps with new schedules of administration that may more effectively expedite pleural drainage in loculated MPE.

## 6. Current Forms of IPFT Used in Loculated MPE and Limitations Regarding Their Use

While several agents; two chain urokinase, tissue plasminogen activator (tPA) and streptokinase have been used to treat pleural loculation in MPE, the doses/dosing schedules that are now used remain empiric, as is the case in loculated pleural infections [45]. It is conceivable that one form of IPFT may be better than another, so the choice of optimal agents remains unclear at present. For example, tPA is rapidly inactivated by plasminogen activator inhibitor-1 (PAI-1) in exudative pleural effusions, while agents that are relatively resistant to this inactivation, such as single-chain urokinase, could theoretically be of advantage. Whether adjuncts such as DNase are advantageous in MPE also remains unclear. Comparative assessments of the safety and efficacy of any of these agents in dose-ranging studies have not, to our knowledge, been reported in MPE. This remains an important gap in the field, but one that persists in the area of pleural interventions for patients with pleural loculation and failed drainage attributable to loculated pleural infections or retained hemothorax [45,46]. It may be that different, well-tolerated doses and possibly more effective dosing and dosing schedules of IPFT could be identified in MPE and other forms of pleural loculation with failed drainage. In the MIST-2 trial, for example, could a dose of fifty percent more tPA (or DNase) have been more effective and as safe versus the 10/5 mg dosing that was used [33]? What was the predicate for delivering these agents twice daily and for three days? The same considerations apply to the interpretation of available studies in patients with pleural loculation and impaired drainage complicating pleural infections, retained hemothorax and MPE. These questions have yet to be answered, but dose reduction in the tPA component of tPA/DNase IPFT has been tested in pleural infections, with evidence of safety and efficacy, so that the authors suggest a 5 mg dose of tPA to be the starting dose in further larger trials of this approach [47]. Whether stratified dosing of IPFT+/− adjuncts would be of advantage in patients with different forms of MPE is unknown, nor is the applicability of this approach clear in patients with varying co-morbidities including bleeding diatheses that may complicate MPE. It is conceivable that tumor burden and/or tumor type could also affect the safety or efficacy of IPFT, representing another area for further study.

## 7. Caveats in the Search for Optimized IPFT in MPE via Clinical Trial Testing

Comparative prospective studies of one fibrinolytic agent versus another in MPE would require considerable logistic support and cost. If comparison of a new form of IPFT with an existing form of IPFT is envisioned, a further challenge could be the requirement for separate investigational new drug (IND) applications; one for each agent, to obtain approval by the US Food and Drug Administration (FDA) to perform a comparative clinical safety trial. While such trials, followed by comparative efficacy trials would be desirable, they may be untenable for logistic and financial reasons. It may be that well organized pleural disease networks as exist in The United Kingdom or Australia are best positioned to conduct such trials, although networks could be established in the US in future. A less cumbersome approach could be the expansion of dosing formats derived from the smaller trials and case reports, as described above. Commercialization of new IPFT agents does not require initial comparative assessments, but rather successful safety and early efficacy trials that may be funded by company sponsors or by agencies such as the National Institutes of Health or the National Cancer Institute in the US.

## 8. The Case for Preclinical Testing of IPFT Designed for Use in Symptomatic Loculated MPE

Preclinical testing in anticipation of clinical trial testing of IPFT (or most other agents) simulates but does not recapitulate actual clinical circumstances. These projects again entail substantial cost and considerable effort, but these factors are offset, in our view, by powerful advantages. Safety or efficacy concerns can be identified early, mitigating the risk of proceeding with marginal or flawed products unlikely to withstand the rigors of clinical trial advancement. Preclinical studies of IPFT dosing can inform the design of clinical dose ranging trials of IPFT, as recently reported [34]. These studies can be used to extrapolate to more precise dosing ranges to be evaluated in initial safety trials. Data from these studies can also strengthen background material which regulatory agencies such as the FDA use to evaluate clinical trial design. The paucity of this type of data in loculated, symptomatic MPE is being addressed by the authors, who are in the process of creating a rabbit model of organizing MPE amenable to interventional testing with different forms of IPFT. This project builds on our experience in creating rabbit models of human chemical injury, empyema at different stages of progression and retained hemothorax [19,48]. These models can also be used to test clinically relevant responses to new forms of IPFT. The clinically relevant responses can be evaluated by imaging including CT scans and renditions and chest ultrasonography. The effects of the IPFT agents on a range of cellular and biochemical responses can similarly be assessed [44]. We envision being able to deploy all of these modalities in a forthcoming model of MPE. We acknowledge that iterations and new schedules of IPFT may be testable in clinical trials without precise supporting preclinical data. However, the currently used IPFT agents to relieve failed drainage are all off-label for loculation in MPE, empyema or retained hemothorax. In our view, preclinical testing represents a safer path to identify optimal forms of new IPFT for clinical trial testing that are most likely to be best tolerated and effective in patients with organizing MPE with failed drainage.

## 9. New Lines of Investigation and Potential Applications in Organizing MPE

The development of a rabbit model of organizing, malignant effusion could reveal underlying mechanisms and novel interventions for MPE. Additionally, further research comparing the pleural tissues and effusions from MPE patients with other exudative effusions—such as those associated with empyema, retained hemothorax, or other forms of pleural organization—could provide valuable insights. These effusions could also be compared with benign transudative effusions, such as those associated with congestive heart failure. New approaches being applied include cellular and molecular profiling of MPE samples that suggest poor prognosis [49,50]. These analyses could be adapted to define prognostic criteria associated with loculation of MPE or even the response to IPFT. Assessment of levels of soluble urokinase receptor or plasminogen activator inhibitor-1 (PAI-1) have been associated with requirements for invasive management of mortality and loculation, respectively, in pleural infections [51,52]. Analyses of these biomarkers could likewise be assessed in MPE to determine if these factors guide invasive treatment of MPE or if reliable prediction of responses to IPFT can be achieved. In an alternative approach, the fibrinolytic potential assay; FPA, an assessment of the bioactivity of fibrinolytic agents in pleural fluids, could potentially guide the personalized use of IPFT dosing in patients with loculated MPE [19]. In the area of combined adjunctive IPFT, suppression of PAI-1 activity could enable lower doses of fibrinolytic agents to be used, which may be of special advantage for patients with loculated MPE and higher bleeding risk [53]. The safety and efficacy of this approach requires future clinical trial testing. High pleural fluid viscosity in malignant mesothelioma may rely, at least in part, on high levels of hyaluronic acid [54] and its role in the pathogenesis of loculated MPE in a broader range of malignancies remains to be studied. Profiling of the miRNAs of a small series of lung and breast cancer MPEs has been performed [55], and the technique could be potentially more broadly used to define new biomarkers of loculation or response to therapy in loculated MPE in the future. Lastly, the profibrogenic capacity of resident pleural mesothelial cells undergoing mesenchymal transition (MesoMT) could play a potentially important role in the formation of adhesions in loculated MPE, an area that requires additional investigation. Key contributors and effectors involved in this profibrogenic phenotypic transition include myocardin, ZIP kinase, Tuftelin 1, and signaling intermediates such as mTORC2. These elements play significant roles in the initiation and progression of MesoMT and represent potential targets for focused investigation in the context of loculated MPE [56,57,58,59].

## 10. Conclusions

Defining the complexities of organization in the pathogenesis of MPE remains a substantial challenge. This underscores the urgent need for investigation of novel therapeutic targets that could enable better treatment strategies in future. Identification of new forms of targeted therapy that are clinically actionable, safe, and most effective could be used to refine IPFT or otherwise independently disrupt the progression of organization in MPE. Advances in biomarker research and personalized approaches such as the FPA could also potentially improve patient outcomes, contingent upon successful future clinical trial testing. Additional scrutiny of the pathogenesis of MPE therefore offers a promising opportunity to improve the quality of life for individuals affected by MPE.

## Data Availability

Data Availability Statement: No new data were created or analyzed in this study.

## Abbreviations

MPE: Malignant pleural effusion. IPFT/IET: Intrapleural fibrinolytic or intrapleural enzymatic therapy. IPC: Indwelling pleural catheter. Urokinase: Urokinase plasminogen activator (uPA). tPA: Tissue plasminogen activator. PAI-1: Plasminogen activator inhibitor-1.
